# Action of Quinine

**Published:** 1864-06

**Authors:** J. Hughes Bennett


ACTION OF QUININE.—That quinine is a tonic I have long had reason to doubt. Quantities of this valuable drug appear to me to be annually wasted in administering it in all sorts of affections as a simple means of increasing appetite or communicating strength. I consider it a vegetable alkaloid, which, like others of its class, operates on special parts of the nervyus system through the blood-vessels. Why it does so we are ignorant. That vegetable poisons do possess this influence constitutes an ultimate fact in the science of therapeutics. But in a similar manner to that in which opium operates on the brain, strychnine and hemlock on the spinal cord, and other drugs operate on other parts of the nervous system: so, I believe, quinine acts on the ganglionic system of nerves, controllng and diminishing those phenomenas of fever which physiology has proved to be produced by their irritatiod or injury.—(*J. Hughes Bennett*) *Lancet.*

